# Exploring the impact of orthodontic appliances on the oral microbiome and inflammatory parameters

**DOI:** 10.1186/s40510-025-00560-8

**Published:** 2025-04-07

**Authors:** Michael Nemec, Patrick Ringl, Kathrin Spettel, Lisa Schneider, Richard Kriz, Sonia Galazka, Marcus Sedlak, Erwin Jonke, Oleh Andrukhov, Athanasios Makristathis

**Affiliations:** 1 Clinical Division of Orthodontics, University Clinic of Dentistry, Medical University of Vienna, Vienna, Austria; 2 Division of Clinical Microbiology, Department of Laboratory Medicine, Medical University of Vienna, Vienna, Austria; 3 Section Biomedical Science, Health Sciences, University of Applied Sciences FH Campus Wien, Vienna, Austria; 4 Division of Infectious Diseases and Tropical Medicine, Department of Medicine, Medical University of Vienna, Vienna, Austria; 5 Division of Data, Statistics and Risk Assessment, Austrian Agency for Health and Food Safety AGES, Vienna, Austria; 6 Competence Center for Periodontal Research, University Clinic of Dentistry, Medical University of Vienna, Vienna, Austria

**Keywords:** Microbiome, Orthodontics, 16S rRNA sequencing, Saliva

## Abstract

**Background:**

The symbiotic relationship between the oral microbiome and the host immune system is a prerequisite of oral health. Disruptions to this system can be associated with the development of diseases like dental caries. Introducing orthodontic treatments, such as aligners and fixed appliances, might impact this microbial ecosystem. This study evaluated potential changes in salivary microbiome and the level of inflammatory marker myeloid-related protein 8/14 in patients undergoing orthodontic treatment with aligners or fixed appliances.

**Methods:**

Forty-eight patients were divided into two groups for treatment with fixed appliances or clear aligners. Unstimulated saliva samples were collected at baseline, three, and six months for microbiome analysis via 16 S rRNA sequencing and MRP-8/14 level measurement using ELISA.

**Results:**

Among 503 identified microbial species, no significant changes were noted in overall microbiome. A considerable increase of caries-relevant species could not be observed either. MRP-8/14 levels remained unchanged across treatments, indicating no alterations in the inflammatory level.

**Conclusion:**

Orthodontic treatment with fixed or removable appliances does not significantly alter the salivary microbiome or influence inflammation, suggesting that these interventions are unlikely to affect oral health negatively.

**Supplementary Information:**

The online version contains supplementary material available at 10.1186/s40510-025-00560-8.

## Introduction

Microorganisms colonizing the human body are essential to our well-being and directly affect various physiological processes like nutritional uptake, metabolism, and host immunity. Each colonization site provides a different niche and harbours its unique microbiome [1, 2]. With advancements in next-generation sequencing, 16 S rRNA targeted sequencing has become a reliable method to comprehensively study these bacterial communities on a species level, opening new possibilities for diagnostic and research purposes [3–5]. The oral cavity is the habitat of the second most complex microbiome in humans and is especially species-rich and diverse, with over 700 species identified to this day [6]. Depending on location, the bacterial composition varies drastically with biofilms on the supra- or subgingival surfaces of teeth, plaques on tooth surfaces or oral mucosa, each inhabiting a specialized set of microbiotas [7, 8]. Saliva has no own resident microbiome and includes the bacteria continuously shedding from the different oral surfaces [9]. While usually benefiting the host, in a dysbalanced state caused by ecological stress, alterations in microbiota found in the different niches of the mouth can lead to the progression of two most common oral diseases–caries and periodontitis [10, 11]. Many factors contribute to the composition of microbiota colonizing the various surfaces of the mouth, including diet, oral hygiene, age and activity of the immune system [12].

Orthodontic treatment (OT) can lead to a deterioration of clinical parameters associated with periodontal diseases, including bleeding on probing, plaque index, the development of pockets or gingival recessions and attachment loss [13, 14]. Proper oral hygiene practices can be impeded through brackets, wires, and other components, which could lead to plaque accumulation and subsequent shifts in the microbial communities [15, 16]. To overcome this problem, removable clear aligners, which the patients wear for 22 h a day, can be used as an alternative [15]. Different from fixed orthodontic treatment, aligners can be temporarily removed, which provides better accessibility for oral hygiene procedures [17]. Nevertheless, aligner treatment is also accompanied by temporarily fixed components, such as buttons or attachments [18]. Furthermore, aligners introduce a new kind of surface for bacterial colonization, especially in cracks and the rough inner surface that is in close contact with the gingival margin [19, 20]. Since clear aligner treatment gained vast popularity among orthodontic patients due to advantages in aesthetics and patient comfort, uprising research interest arose upon this orthodontic treatment modality [21]. Recent studies indicate that therapy using clear aligners has minimal impact on periodontal health and the salivary microbial community structure (15,16,22). To date, a superiority of clear aligners over fixed brackets regarding their effect on oral health is suspected, but it has not been proven to be clinically relevant [23]; both treatment modalities are alleged to have a similar impact on the oral health of orthodontic patients [24].

Some previous studies have already investigated the potential alteration of the oral microbiome during different OTs. A study on the supragingival microbiome using 16 S rRNA sequencing showed some differences in the microbial community between fixed brackets and clear aligners [25]. Another study investigated the salivary microbiome and observed dysbiosis in both treatment modalities [26], whereas studies using multiplex PCR imply differences regarding the presence of certain species between orthodontic patients treated with fixed appliances and clear aligners [27]. Therefore, there is still a strong demand for further studies to illuminate the effect of different types of orthodontic treatments on the oral microbiome.

Besides microbiome assessment and clinical parameters, gingival inflammation could be assessed by determining various inflammatory markers [28]. One of these markers is the myeloid-related protein (MRP)-8/14, also known as calprotectin or S100A8/9 [29], which is produced by neutrophils in response to inflammatory stimuli [30]. The local levels of this marker are already increased at the initial stages of gingival inflammation [31, 32]. Therefore, the measurements of MRP-8/14 during various orthodontic treatments could provide additional insight into the inflammatory response [33].

In the present study, we collected saliva and investigated the microbiome and inflammatory status to compare the effect of the orthodontic treatment with either fixed brackets or clear aligners on oral health. Sampling saliva is a promising method as it is an easy and non-invasive procedure and is a convenient source of various biomarkers [34]. We especially focused on a potential shift of the salivary microbiome in patients over six months of therapy with either clear aligners or fixed orthodontic appliances. The periodontal conditions were evaluated by measuring plaque index, probing depth and bleeding on probing. Additionally, we assessed the inflammatory state by measuring salivary level of MRP-8/14.

## Materials and methods

### Test population and sampling

In this longitudinal study, a total of 48 patients were recruited and split according to their received orthodontic treatment option. The first group involved 24 patients wearing Invisalign aligners, while the second group included 24 patients receiving therapy with fixed appliances (brackets).

The following inclusion criteria were considered for the selection of study participants: age ≥ 18 years, skeletal class I, and minimal mandibular crowding (Little Index grade I-III). Exclusion criteria included: currently smoking, chronic diseases, restorations with close contact to the gingival margin, presence of crowns or bridges, previous conservative periodontal treatment (full mouth disinfection, full mouth debridement), medication with antibiotics, steroids or non-steroidal anti-inflammatory drugs within the past three months, pregnancy, and diabetes. Patients were instructed in oral hygiene procedures four weeks before enrolment in the study and had to clean their teeth at least twice daily. Inclusion and exclusion criteria were chosen to minimize the effect of various factors favoring plaque accumulation and oral dysbiosis. Additional evaluation on patients at T1 and T2 has not been documented.

Allocation to the groups depended on the malocclusion and on the patient’s request; therefore, no randomisation was possible. The patients in the bracket group were treated with a buccal self-ligating multibracket appliance of a 0.022-inch slot size (Empower^®^, American Orthodontics, Weil am Rhein, Germany) and aligner patients were treated with orthodontic aligners (Invisalign^®^, Align Technology Inc., Santa Clara, CA, USA). The patients were recruited at the Clinical Division of Orthodontics, University Clinic of Dentistry, Medical University of Vienna. Study visits were planned at three different time points: before treatment (T0), three months after the start of treatment (T1) and six months after the start of treatment (T2). At each visit, whole saliva samples were collected and frozen. Furthermore, the plaque index (PI), probing pocket depth (PPD) and bleeding on probing (BOP) were assessed on six teeth (Ramfjord teeth 16, 21, 24, 36, 41 and 44). Probing depth was measured in six gingival areas: distobuccal, buccal, mesiobuccal, mesiolingual (or mesiopalatinal), lingual (or palatinal) and distolingual (or distopalatinal), and the greatest value was taken for the analysis. Measurements were performed with a periodontal probe (Hu-Friedy, IL, USA) by three different operators with at least three years of clinical experience and extensive training in the procedure. For plaque index measurements, the Plaque Control Record [35] was used as follows: teeth were stained using a foam pellet (Erkodent^®^, Pfalzgrafenweiler, Germany) and a plaque staining agent (Mira-2-Ton, Hager Werken, Duisburg, Germany). Patients were asked to rinse their mouths with water, and a supragingival plaque was recorded. The number of plaque-positive areas was divided by the number of measurement areas and multiplied by hundred.

All patients received oral hygiene instructions prior to treatment, but how strictly they followed these instructions was not recorded. Saliva at T0 was collected for 2 min without stimulation before measuring the periodontal parameters and before the start of the orthodontic treatment. For this purpose, the patients were instructed not to consume any food or drinks except water one hour before sampling. Before collecting saliva, the oral cavity was rinsed thoroughly with distilled water. Collected saliva was aliquoted in 0.5 mL samples, placed at − 80 °C immediately, and stored there until analysis. The periodontal parameters measurements and saliva collection at T1 and T2 were done similarly. Oral hygiene and lifestyle habits were assessed by a questionnaire.

### ELISA MRP-8/14

The MRP-8/14 concentration in saliva samples was assessed using a commercially available MRP-8/14 Sandwich ELISA Kit (Bühlmann Laboratories, AG, Schoenbuch, Switzerland). Dilutions were prepared according to the manufacturer’s protocols previously described [32, 36].

### Microbiome analysis − 16 S rDNA sequencing

DNA extraction was performed according to protocol involving bead-beating steps and chloroform extraction [37, 38]. Resulting DNA quality and quantity were assessed with a NanoDrop 2000c spectrophotometer (Thermo Fisher Scientific, Waltham, MA, USA) and with a Qubit dsDNA High Sensitivity Assay kit (Life Technologies, Carlsbad, CA, USA), respectively [39, 40]. Subsequently, in a broad range PCR the V3 and V4 regions of the ribosomal RNA gene were amplified and sequencing libraries were prepared according to Illumina DNA Prep^®^ Protocol [41]. The library was diluted to a final loading concentration of 4 pM with a 5% PhiX spike (PhiX Control v3, Illumina). Sequencing was performed on an Illumina© MiSeq system (Illumina, San Diego, CA, USA) with a v3 flow cell.

### Bioinformatic analysis

Sequencing results were processed using amplicon sequence variant (ASV)-based Divisive Amplicon Denoising Algorithm 2 (DADA2) [42]. This algorithm only aligns reads with 100% sequence identity to database entries. First, nucleotides like primers and adaptors were trimmed. Then DADA2 produced a list of all ASVs and the number of times they were present in each sample [43]. The generated sequences were compared with the SILVA ribosomal RNA database and assigned to the corresponding taxonomy [44]. Inter- and intragroup diversities were analysed using *phyloseq* [45].

### Statistical analysis

Numeric data is reported as *mean (standard deviation [SD])* for normally distributed continuous variables and median *(interquartile range [IQR])* for non-normal continuous and discrete variables. Categorical variables are reported as *count (ratio [%])*. Normal distribution was confirmed by Shapiro-Wilk test. Differences in demographic characteristics, oral hygiene and lifestyle habits were assessed by Mann-Whitney-U test or Fisher’s Exact test. The interaction of therapy method (between-subject factor) and time (within-subject factor) on clinical parameters was assessed for MRP-8/14, PI and PPD using mixed-design analysis of variance (mixed ANOVA). Depending on the ANOVA results, Welch t-test for unequal variances to compare the therapy methods at each time point or paired t-test to compare the time points within each group were performed. Differences in BOP between therapy groups were assessed at each time point using the Mann-Whitney-U test. Changes in BOP over time were assessed for each therapy group using multiple Wilcoxon signed-rank tests for paired data. P-values were adjusted for multiple tests using the Holm correction.

To determine the intragroup diversity, or alpha diversity, the Chao1-, Shannon- and Simpson indices were calculated. The intergroup, or beta diversity, was determined using Bray-Curtis based non-metric multidimensional scaling (NMDS).

Relative abundance (%) was assessed descriptively. Differential abundance between therapy groups at each time point is demonstrated using volcano plots. The log2-foldchange was calculated as: $\:FC=\:log2\left({abundance}_{Bracket}\right)-log2\left({abundance}_{Invisalign}\right)$. Taxon abundances in the therapy groups were compared using Welch t-tests and correcting for multiple testing using the Benjamini-Hochberg procedure.

Statistical analyses were performed using R (version 4.3.1, R Core Team 2023, Vienna, Austria) and the packages *rstatx*, *tidyverse*,* ggsci*,* ggpubr* and *phyloseq*.

## Results

### Patient characteristics and clinical data

Demographic characteristics, oral hygiene and lifestyle habits of patients are presented in Table 1. Three patients with bracket and one patient with Invisalign treatment dropped-out at the time point T1. At the time points T0 and T2 the data were collected from all participants.

**Table 1:** Demographic characteristics, oral hygiene and lifestyle habits of patients

	Bracket T0	Bracket T1	Bracket T2	Invisalign T0	Invisalign T1	Invisalign T2
Participants	24	21	24	24	23	24
Sex: female, count (%)	11 (45.8)	9 (42.9)	11 (45.8)	15 (62.5)	14 (60.9)	15 (62.5)
Age, median (IQR)	28 (12.5)	28 (12)	28 (12.5)	26.5 (14.25)	26 (13)	26.5 (14.25)
Sugary beverages per day, median (IQR)	0 (1)	0 (0.5)	0 (1)	0 (1)	0 (1)	0 (1)
Meals per day, median (IQR)	2.5 (1)	2.5 (1)	3 (1)	2 (1)	3 (0.88)	2.5 (1)
Snacks per day, median (IQR)	2 (2.12)	2 (2)	1.75 (1.62)	2 (0.5)	1 (2)	1 (0.5)
Uses mouthwash, count (%)	12 (50)	13 (61.9)	16 (66.7)	4 (17.4)	7 (31.8)	7 (30.4)
Mouthwash per week, median (IQR)	0.5 (7)	3.5 (7)	6.5 (11.38)	0 (0)	0 (3.38)	0 (3)

IQR (interquartile range) represents the range between the first (25th percentile) and third (75th percentile) quartiles of a dataset, thus encompassing the middle 50% of the data points

In the initial assessment of our cohort, the Bracket group at T0 consisted of 24 participants, of which 45.8% (*n* = 11) were female, with a median age of 28 years (IQR = 12.5).

Comparatively, the Invisalign group at T0 also comprised 24 individuals, with a higher proportion of females at 62.5% (*n* = 15). For T0, there were no significant differences in the assessed parameters between the Invisalign and fixed appliance groups, except for use of mouthwash (*p* = 0.030) and the frequency of mouthwash use per week (*p* = 0.014).

**Fig. 1:**
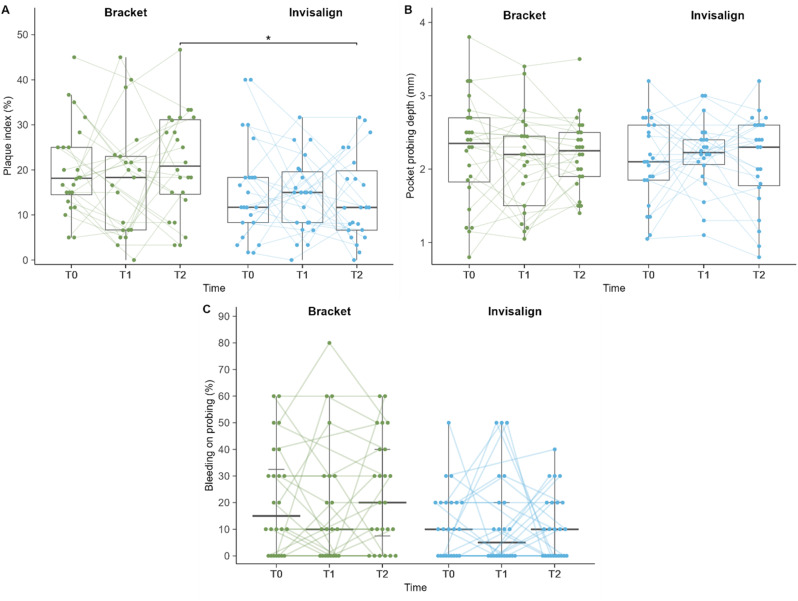
Plots of clinical parameters over time by treatment; Dots indicate individual participants. Lines indicate trajectories of parameters over time. (**A**) Boxplot of PI [%], * statistically significant difference at T2 by Welch t-test (*p* = 0.040); (**B**) Boxplot of PPD [mm]; (**C**) Dot plot of BOP [%], horizontal lines indicate median (bold line) and interquartile range, vertical lines indicate the range excluding outliers

Plots of clinical parameters over time by treatment are shown in Fig. 1. Using mixed ANOVA a significant difference of the PI was found across all sampling time points combined between the two therapy groups (*p* = 0.026). Over all three time points combined, patients who received bracket therapy had higher mean PIs (19.82 [SD = 11.17]), compared to those receiving Invisalign therapy (14.54 [SD = 9.68]). This was confirmed by a post-hoc t-test (*p* = 0.004).

However, the model indicated that the PI did not change significantly over time within groups (*p* = 0.605). When assessing the time points individually, higher mean PIs were observed at all time points in the bracket group (T0 = 20.04 [SD = 9.90], T1 = 17.67 [SD = 12.56], T2 = 21.49 [SD = 11.25]), compared to the Invisalign group (T0 = 15.65 [SD = 11.22], T1 = 14.30 [SD = 8.33], T2 = 13.67 [SD = 9.54]), with only the difference at T2 being statistically significant (*p* = 0.040).

Also the PPD did not change significantly over time within groups (*p* ≥ 0.822) and no significant difference in the mean PPD between the brackets (T0 = 2.25 [SD = 0.74], T1 = 2.10 [SD = 0.68], T2 = 2.21 [SD = 0.48]) and the Invisalign group (T0 = 2.12 [SD = 0.58], T1 = 2.20 [SD = 0.47], T2 = 2.12 [SD = 0.63]) was observed at any of the time points (*p* = 1).

Median BOP was similar in the brackets (T0 = 15.0 [IQR = 32.5], T1 = 10.0 [IQR = 30.0], T2 = 20.0 [IQR = 32.5]) and Invisalign group (T0 = 10.0 [IQR = 20.0], T1 = 5.0 [IQR = 20.0], T2 = 10.0 [IQR = 20.0]) at each of the time points (*p* ≥ 0.345). BOP did not change significantly over time within each group (*p* = 1).

### MRP-8/14

In the MRP-8/14 analysis, five values above 19 µg/ml deviated significantly from the other values. These five values were confirmed as outliers using the IQR-criterion and were therefore excluded from the analysis. After exclusion of outliers, MRP-8/14 was still heavily right skewed. Therefore, the variable was log-transformed to achieve normal distribution for analysis. Original and transformed measurements are reported in Table 2.

**Table 2:** Median MRP-8/14 and mean log transformed MRP-8/14 values (µg/ml)

	Bracket T0	Bracket T1	Bracket T2	Invisalign T0	Invisalign T1	Invisalign T2
MRP-8/14 µg/ml, mean (SD)	2.03 (1.13)	1.97 (1.26)	2.36 (1.95)	1.6 (1.03)	2.33 (2.38)	1.98 (1.66)
Log-MRP-8/14, mean (SD)	0.52 (0.77)	0.48 (0.65)	0.59 (0.76)	0.26 (0.71)	0.42 (0.96)	0.44 (0.68)

**Fig. 2:**
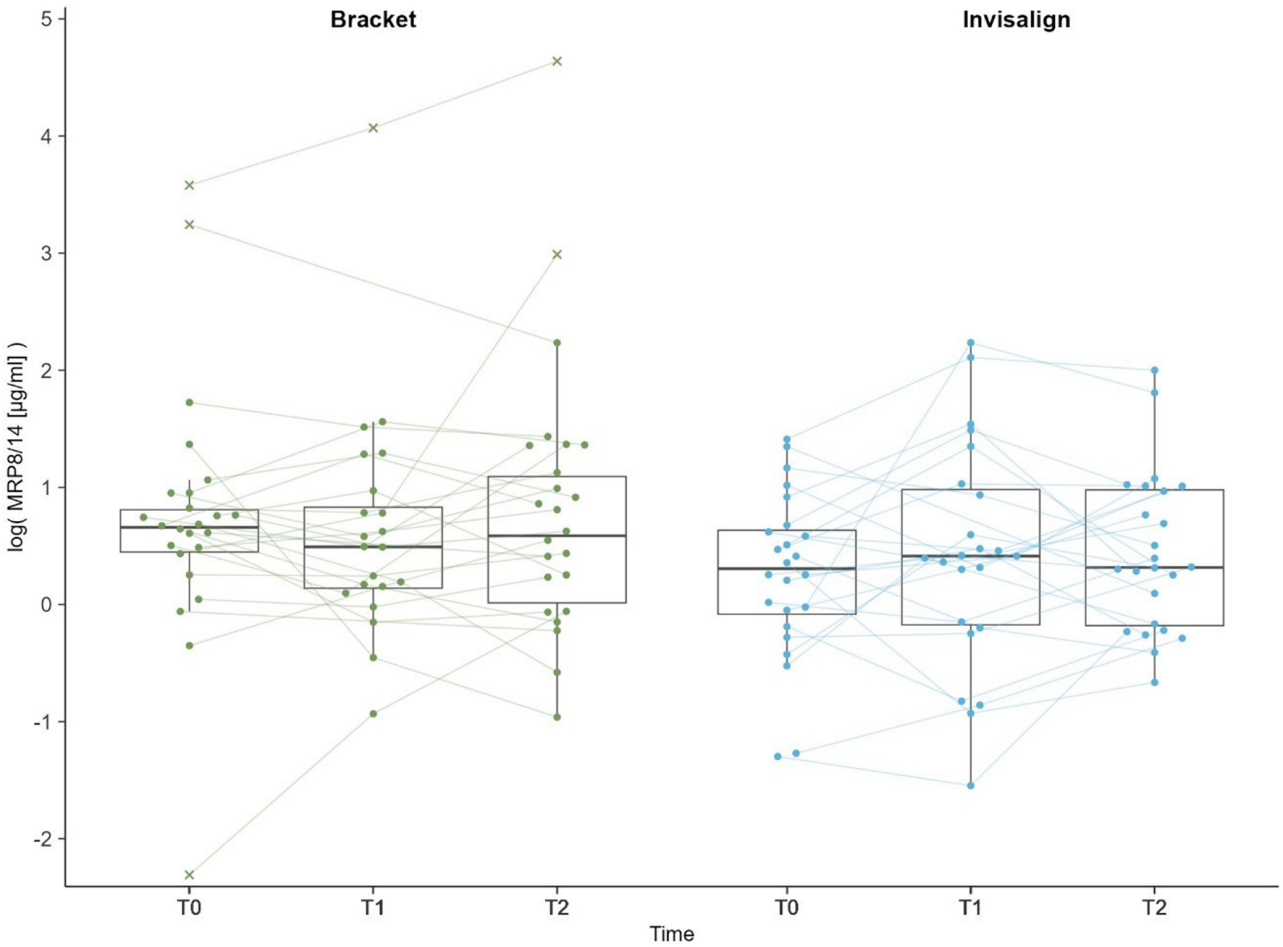
Boxplot of log transformed MRP-8/14 (µg/ml) over time by treatment; Dots indicate individual participants. X indicates outlier values that were excluded from the analysis. Lines indicate trajectories of log transformed MRP-8/14 levels over time

Mixed-ANOVA did not indicate a significant interaction of therapy and time on the log-transformed levels of salivary MRP-8/14 (*p* = 0.138; Fig. 2). No significant difference in the mean log-MRP-8/14 values was observed between the bracket and Invisalign group at any time point (*p* ≥ 0.726, Table 2). Log-MRP-8/14 did not change within group over time (*p* ≥ 0.559).

### Sequencing results

#### Alpha- and beta diversity

**Fig. 3:**
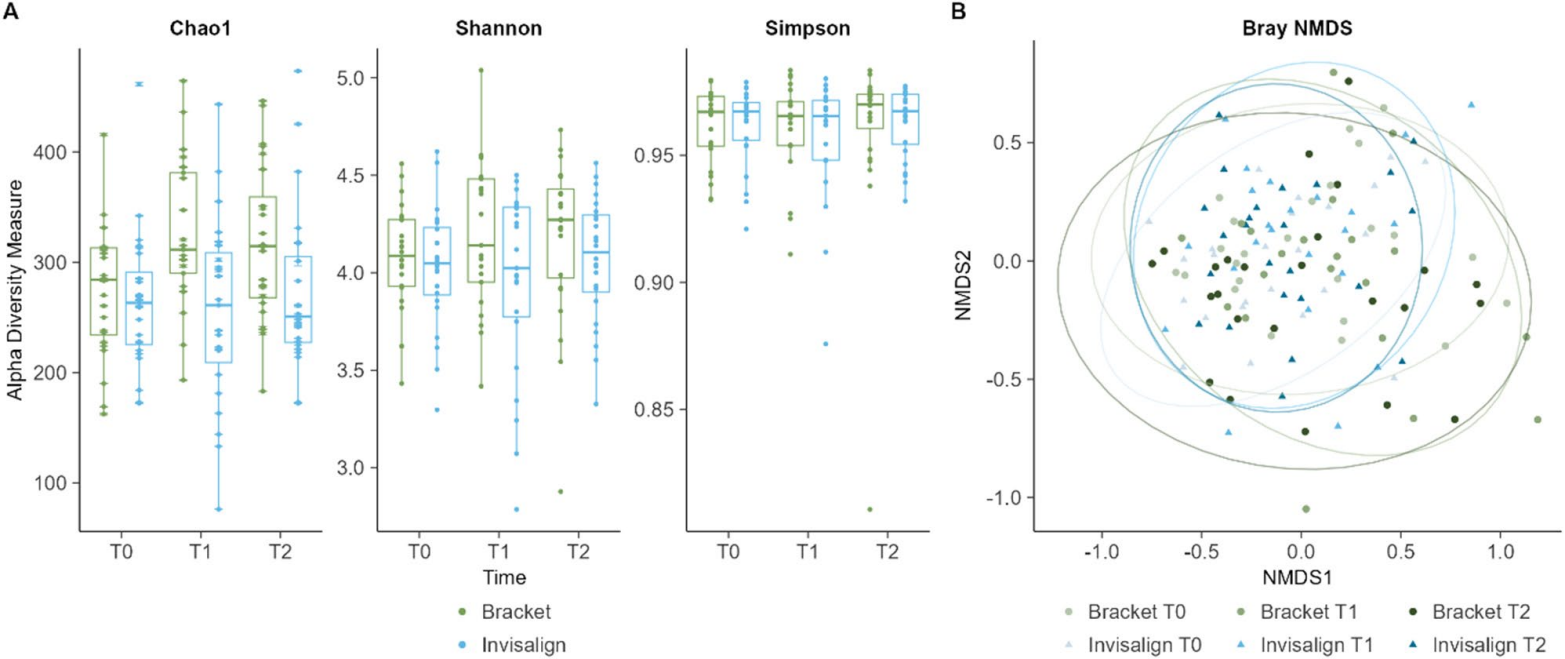
Alpha- and beta diversity results of salivary microbiome; **A**) Box plots for Chao1-, Shannon- and Simpson-Index for bracket and Invisalign group at T0, T1 and T2. **B**) Bray NMDS Plot of all samples at T0, T1 and T2

The intragroup diversity, or alpha diversity, was calculated using Chao1-, Shannon and Simpson indices (Fig. 3.A). All three diversity indices of the Invisalign group at T0, T1 and T2 remained unchanged, with no significant differences detected. An increase of alpha diversity measure can be noted for the bracket group at T1 and T2 in the Chao1 and Shannon index. However, neither the differences between time points nor between the groups were statistically significant. Further investigation of the community diversity was done by beta diversity analysis using a Bray-Curtis-based NMDS plot of all samples. No clear clustering between bracket and Invisalign groups was observed (Fig. 3.B). Furthermore, neither group showed a clear separation between T0, T1 and T2. This indicates that the microbial community profile remained stable over the course of the therapy.

#### Composition of the oral Microbiome

**Fig. 4:**
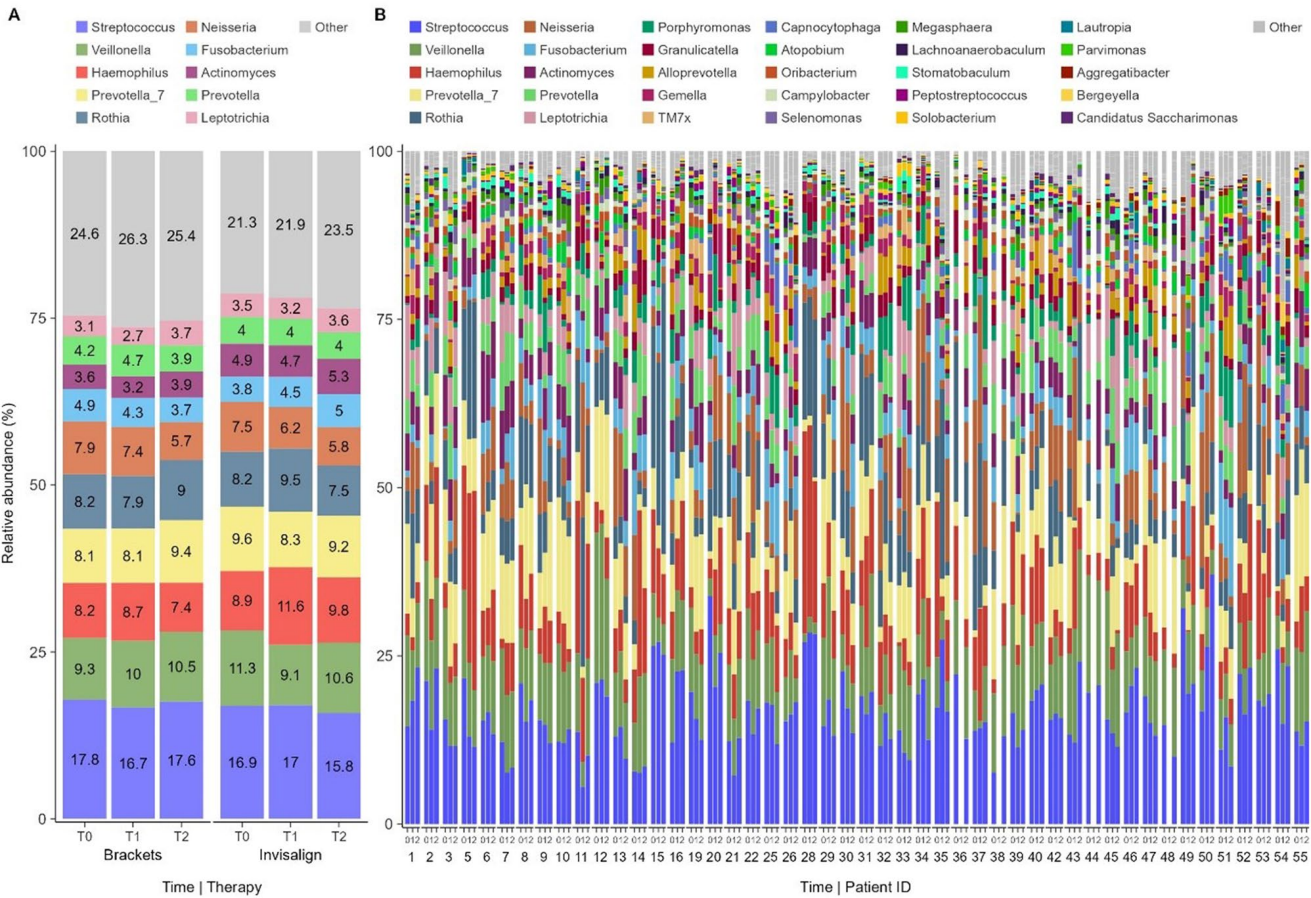
Descriptive depiction of relative abundance; **A**) Percentage of relative abundance of ten most abundant genera shown at T0, T1 and T2 in brackets and Invisalign group. **B**) Most abundant genera and their relative abundance at T0, T1 and T2 for each patient individually. Other includes less abundant genera and unidentified ASVs

A total of 503 distinct species from 201 genera were identified from 140 samples. The ten most abundant genera were *Streptococcus*,* Veillonella*,* Prevotella_7*,* Rothia*,* Haemophilus*,* Neisseria*,* Fusobacterium*,* Prevotella*,* Actinomyces*, and *Leptotrichia* (Fig. 4.A). Together, they made up 76.2% of all reads (73.7-78.7%, depending on the group). The top ten genera were the same between the therapy groups, but with slight differences in their relative abundances. There was a substantial decrease in the relative abundance of *Neisseria* over time at T2 compared to T0 in both groups.

Volcano plot analysis revealed no statistically significant differences in relative abundance of the detected taxa at any of the time points between groups (Supplemental Fig. 1) or between different time points within a group.

**Fig. 5:**
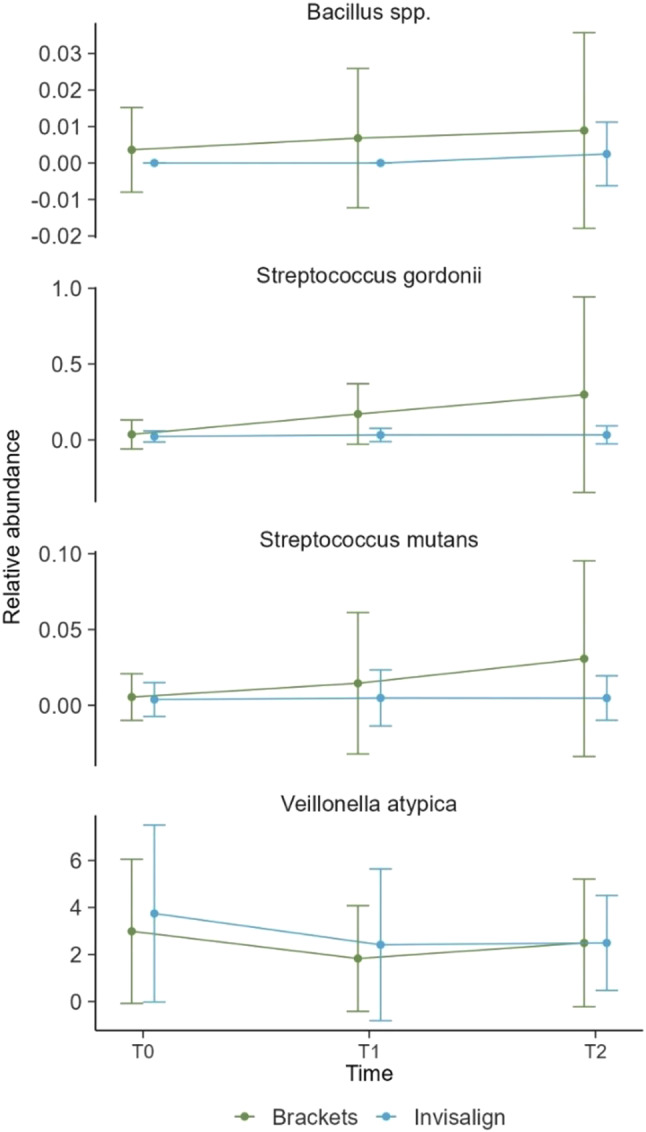
Variations in the relative abundance of caries-relevant bacterial species; Depicted in the figure are relative abundance of *Bacillus spp.*,* Streptococcus gordonii*,* Streptococcus mutans*, and *Veillonella atypica*—over the three time points (T0, T1, and T2). The data from brackets (green lines) and Invisalign (blue lines) are depicted separately. Error bars indicate standard deviations from the mean

Further analysis was performed of caries-relevant bacterial species including *Bacillus spp.*,* Streptococcus gordonii*,* Streptococcus mutans*, and *Veillonella atypica* (Fig. 5). For *Bacillus spp*., there is an unsubstantial increase in relative abundance from T0 to T2 for the brackets group while levels in the Invisalign group remained close to zero. In the case of *Streptococcus gordonii*, the relative abundance remains low for the Invisalign treatment across all time points, while a slight continuous increase is observed in the brackets group. Similarly, the relative abundance of *Streptococcus mutans* exhibits a slight increase from T0 to T2 in the bracket group. For *Veillonella atypica*, both groups exhibit a marked decrease at T1 followed by a return to near initial levels at T2. None of the observed changes was deemed significant.

## Discussion

This longitudinal study investigated potential changes in the salivary microbiome and assessed the concentration of the biomarker for inflammation MRP-8/14 in 48 patients receiving orthodontic therapy. The aim of this study was to draw a comparison between clear aligner treatment (Invisalign) and fixed appliances and determine whether there are significant differences over the course of the first six months of therapy.

Treatment with fixed orthodontic appliances has the potential to deteriorate oral health by impeding the ability to perform oral hygiene, resulting in plaque accumulation [13, 14]. It has been investigated whether orthodontic treatment using clear aligners results in better periodontal health and less retention of plaque when compared to fixed appliances [46]. Interestingly, in our study the cohort of patients receiving fixed appliances increased the reported frequency of weekly mouthwash use drastically (Table 1).

This study revealed no significant change in all three clinical parameters, PI, PPD, and BOP, over the course of six months in either treatment group. The fact that no deterioration of all clinical parameters was identified within the groups leads to the conclusion that neither treatment option had a significant impact on oral health within the observation period. It should be noted that considerable differences especially in PI were observed between the two treatment groups starting from T0. However, these failed to reach statistical significance at T0 and T1, likely due to our limited sample size. Higher values of BOP were also observed in patients treated with brackets compared to Invisalign already before the start of treatment.

The treatment choice was based on the clinical indication and the patient’s wish, and, therefore, no randomisation was possible. However, a lack of significant differences over time suggests that patients’ oral hygiene practices were sufficient to manage plaque accumulation effectively in both groups. It should be mentioned that it was impossible to control how strictly the patients followed oral hygiene instructions, which might have a certain impact on the study output.

MRP-8/14 is considered as a potential marker of inflammation because its level in saliva and gingival crevicular fluid is enhanced not only in periodontitis but also potentially in gingivitis [31, 47–49]. Furthermore, there is evidence that elevated MRP-8/14 levels are observed in children undergoing bonded maxillary expansion treatment [50]. However, in our study, we did not find any differences in the salivary MRP-8/14 levels, suggesting no alteration in periodontal inflammation over the course of the therapy with both treatment modalities. It is important to note that there are several other potential inflammatory markers, such as CRP, cytokines or metalloproteinases that could also be influenced by orthodontic treatment [49, 51]. A recent study showed that orthodontic treatment with labial and lingual fixed orthodontic appliance is accompanied by increased salivary levels of interleukins IL-1β, IL-6, IL-17 A and monocyte chemoattractant protein 1 [52]. Future studies could address the effect of various orthodontic treatments on the level of these markers, as well as other potential markers.

There was a clear separation of MRP-8/14 values above 19 µg/ml (19.85, 25.63, 35.87, 58.54, 103.44), compared to the rest of the data. These high levels all occurred in the bracket group at various time points with the three highest being from the same subject (Patient 51). While MRP-8/14 occurs generally higher in saliva, with one study indicating an average concentration of around 16 µg/ml, higher values of above 25 µg/ml were normally associated with periodontitis [47]. It is possible that this subject had an inflammation prior to the start of therapy and any increase or decrease would be independent of OT. Therefore, 19 µg/ml was used as a cutoff and values above were confirmed to be outliers by the IQR-criterion and excluded from the analysis. Sensitivity analysis including the previously excluded outliers showed no significant two-way interaction of method and time in the MRP-8/14 levels (*p* = 0.532). Therefore, sensitivity analysis did not indicate an influence of the removal of outliers on the results.

Several sources reflect the microbial communities present in the oral cavity, including saliva, subgingival-, supragingival and dental plaque [7–9]. In this study, we opted to use saliva samples from patients. Saliva is abundant and easily accessible in the orthodontic clinic, making it a practical choice for sample collection. Furthermore, while swab samples and dental plaque provide information about specific microbial composition at localized sites on the teeth, the salivary microbiome offers a comprehensive view of overall oral microbiota because it includes bacteria shedding from all oral surfaces [9, 53]. However, it should be noted that using saliva as a sample source may not effectively capture site-specific alterations in the oral microbiome.

As shown in Fig. 3, the NMDS plot reflecting the beta diversity showed no distinct clustering between the two treatment options or the different time points. A closer look at the MRP-8/14 outliers with high inflammatory levels also showed no distinct clustering in NMDS plot and unremarkable alpha diversity changes (not shown). Despite the overall absence of statistically significant microbiome differences in our study, we observed a tendency for higher alpha diversity values, as indicated by Chao1 and Shannon indices, in the Bracket group at both T1 and T2 compared to the Invisalign group. This increase could potentially result from microbiota changes in specific oral niches that are reflected in the saliva. While we were unable to test this hypothesis, a study by Kado et al. supports this assumption. They detected an increased diversity in supragingival communities while no considerable shifts in salivary microbiome was observed in patients treated with fixed orthodontic appliances [54].

In agreement to our study, Zhao et al. investigated the salivary microbiome in the aligner patients prior to treatment and at six months of follow-up and found no difference in alpha diversity or beta diversity between these two time points [22]. In a cross-sectional study, Wang et al. investigated clear aligner patients (Invisalign system), fixed appliances and healthy controls in a smaller sample size (*n* = 5 per group). Contrary to us, their findings displayed a dysbiosis of the salivary microbiome in both groups of orthodontic treatment, indicating possible disadvantages regarding oral health by orthodontic treatment in general [26]. Previous studies on tooth-associated microbiomes were not conclusive either. A longitudinal study by Shooken et al. found no difference in the alpha diversity of subgingival plaque between patients with brackets and aligners [25]. Guo et al. also investigated the alteration of the subgingival microbiome in female patients with fixed appliances within the first three months after beginning the therapy and found no significant changes in the alpha diversity, however, a non-significant increase in the abundance of some periodontitis-associated bacteria [55]. In contrast, a recent cross-sectional study by Zheng et al. found an increased alpha-diversity in the supragingival plaque of patients wearing brackets for at least 6 months compared to patients treated with Invisalign for the same time period [56]. Several factors contribute to these contrary results including the sampling site, study designs, sample size, oral hygiene instructions, and compliance with oral hygiene instructions by patients. Future research with larger sample sizes and combined analyses of site-specific and salivary microbiomes will be crucial to clarify these relationships.

In Supplemental Fig. 2 the genera with highest differences are depicted between Brackets, Invisalign and MRP-8/14 outliers of the Bracket group. Genera like *Fusobacterium*, *Porphyromonas*, *Parvimonas* and *Alloprevotella* are slightly increased at all time points in the outlier group while *Rothia* and *Haemophilus* are decreased. No substantial differences can be observed between Invisalign and Bracket groups further suggesting that differences in the outlier group occurred before and independent of Bracket or Invisalign treatment.

Deeper analysis of bacterial species associated with development of dental carries revealed a slight, yet statistically insignificant increase of *S. gordonii* and *S. mutans* in the bracket group [57]. However, it might be interesting to analyse whether this is a trend that extends beyond the time frame of this study. Furthermore, no increased relative abundance was found for species that are usually associated with periodontitis and may also play a role in gingivitis (Supplemental Fig. 3). This further suggests the safety of both treatment options for oral health over the course of 6 months.

Taken together, our study population can be described to have throughout the treatment a good oral hygiene. Clinical, microbial and inflammatory parameters suggest non-disruptive oral conditions. Other studies reporting deterioration of periodontal parameters also described deteriorations of the oral microbiome [20, 25].

The major limitations of this study are the use of salivary samples for microbiome analysis, which does not precisely reflect the local microbiomes at different oral surfaces. Orthodontic treatment, especially fixed appliances, partially impede oral hygiene, which might result in plaque accumulation on teeth. Therefore, investigating the site-specific changes in the microbiome, particularly in supra- and subgingival plaque, could provide valuable additional information [13–16]. The missing sample size analysis may be a further limitation. However, it has been difficult to define the microbial community parameters to be used for this calculation, partly due to the discrepancy in the published data. Another limitation is the lack of documentation of follow-up on treatments including use of antibiotics or anti-inflammatory drugs at T1 and T2. Furthermore, most orthodontic treatments extend much longer than six months, thus, a long-term change in the microbial profile cannot be ruled out with this study. A study designed to follow patients entirely throughout the therapy and analyse further parameters for a longer time period may provide more substantial and accurate evidence. This study included only adult patients and, therefore, does not apply to children and minors.

In conclusion, we showed that six months of therapy in patients with good oral hygiene with either fixed or removable orthodontic appliances did not have any significant effect on the salivary microbiome. No quantitative or qualitative shift could be observed. The presence of putative caries or periodontal pathogens did not increase either. Furthermore, the investigation of the inflammatory biomarker MRP-8/14 in saliva and clinical periodontal parameters suggest that oral health has not deteriorated in orthodontic patients during the study period.

## Electronic supplementary material

Below is the link to the electronic supplementary material.

Supplemental Fig.1. Volcano plot indicating the differential abundance of bacterial species in bracket or Invisalign therapy; (A) T0, (B) T1 and c) T2. The significance cutoff, indicated by the horizontal line, is set to *p* = 0.05, the cutoff for the log2-foldchange is set to one, indicated by the strong vertical lines. Species passing one of the cutoffs are labelled. Right side of the graphs depicts species more abundant in Bracket group compared to Invisalign on the left side.

Supplemental Fig.2. Relative abundance (%) of the top ten genera with the highest differences; Depicted are the groups Bracket, Bracket (Outliers) including information of patients excluded from MRP-8/14 analysis, and Invisalign.

Supplemental Fig.3 Variations in the relative abundance of gingivitis-relevant bacterial species; Depicted in the figure are relative abundance of *Fusobacterium nucleatum*,* Parvimonas micra*,* Prevotella intermedia*,* Porphyromonas gingivalis*,* Eikenella corrodens*,* Filifactor alocis*,* Tannerella forsythia* and *Treponema denticola* —over the three time points (T0, T1, and T2). The data from brackets (green lines) and Invisalign (blue lines) are depicted separately. Error bars indicate standard deviations from the mean.

## Data Availability

The sequencing data are available in the BioProject database under accession number PRJNA1234397.

## Abbreviations

OT: Orthodontic treatment
PI: Plaque index
PPD: Probing pocket depth
BOP: Bleeding on probing
ASV: Amplicon sequence variant
SD: Standard deviation
IQR: Interquartile range
NMDS: Non-metric multidimensional scaling
