# Correction: Exploring the impact of orthodontic appliances on the oral microbiome and inflammatory parameters

**DOI:** 10.1186/s40510-025-00577-z

**Published:** 2025-08-21

**Authors:** Michael Nemec, Patrick Ringl, Kathrin Spettel, Lisa Schneider, Richard Kriz, Sonia Galazka, Marcus Sedlak, Erwin Jonke, Oleh Andrukhov, Athanasios Makristathis

**Affiliations:** 1 Clinical Division of Orthodontics, University Clinic of Dentistry, MedicalUniversity of Vienna, Vienna, Austria; 2 Division of Clinical Microbiology, Department of Laboratory Medicine, Medical University of Vienna, Vienna, Austria; 3 Section Biomedical Science, Health Sciences, University of AppliedSciences FH Campus Wien, Vienna, Austria; 4 Division of Infectious Diseases and Tropical Medicine, Department ofMedicine, Medical University of Vienna, Vienna, Austria; 5 Division of Data, Statistics and Risk Assessment, Austrian Agency forHealth and Food Safety AGES, Vienna, Austria; 6 Competence Center for Periodontal Research, University Clinic ofDentistry, Medical University of Vienna, Vienna, Austria

**Correction: Progress in Orthodontics (2025) 26:13**

10.1186/s40510-025-00560-8.

Following publication of the original article [1], the authors identified an error in the author name and e-mail address of Oleh Andrukhov.

The incorrect author name is: Oleh Andruhkov.

The correct author name is: Oleh Andrukhov.

The author group has been updated above and the original article [1] has been corrected.

## References

[1] Nemec M, Ringl P, Spettel K, et al. Exploring the impact of orthodontic appliances on the oral microbiome and inflammatory parameters. Prog Orthod. 2025;26:13. 10.1186/s40510-025-00560-8. 40189709 10.1186/s40510-025-00560-8 PMC11973030

