# Supplementary figures and images for: Exploring the impact of orthodontic appliances on the oral microbiome and inflammatory parameters
Source: Prog Orthod. 2025 Apr 7;26:13. doi: 10.1186/s40510-025-00560-8 (PMC11973030; doi:10.1186/s40510-025-00560-8)

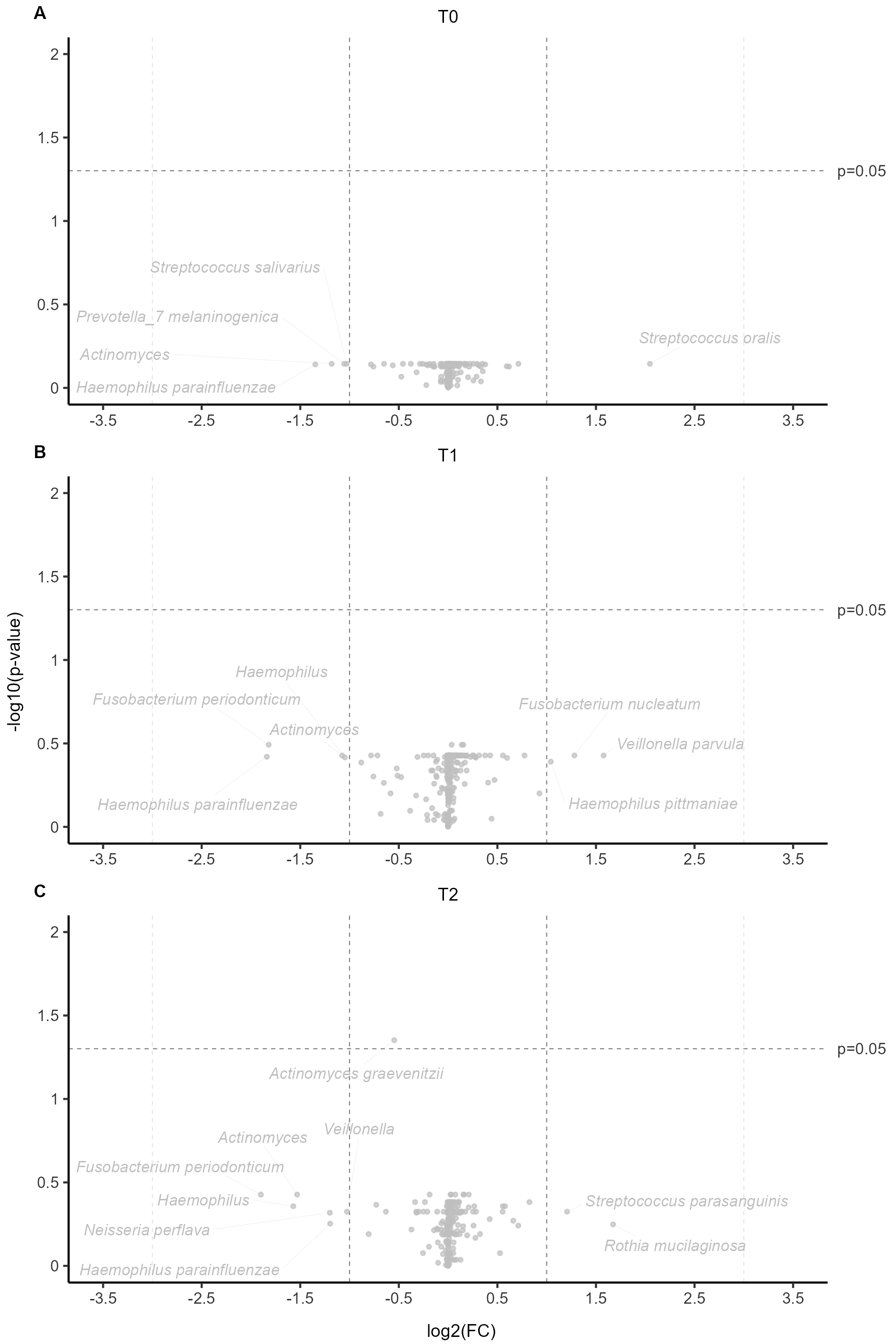

Supplement: Supplementary file 1 — Supplemental Fig.1. Volcano plot indicating the differential abundance of bacterial species in bracket or Invisalign therapy; (A) T0, (B) T1 and c) T2. The significance cutoff, indicated by the horizontal line, is set to p = 0.05, the cutoff for the log2-foldchange is set to one, indicated by the strong vertical lines. Species passing one of the cutoffs are labelled. Right side of the graphs depicts species more abundant in Bracket group compared to Invisalign on the left side. [file 40510_2025_560_MOESM1_ESM.png]

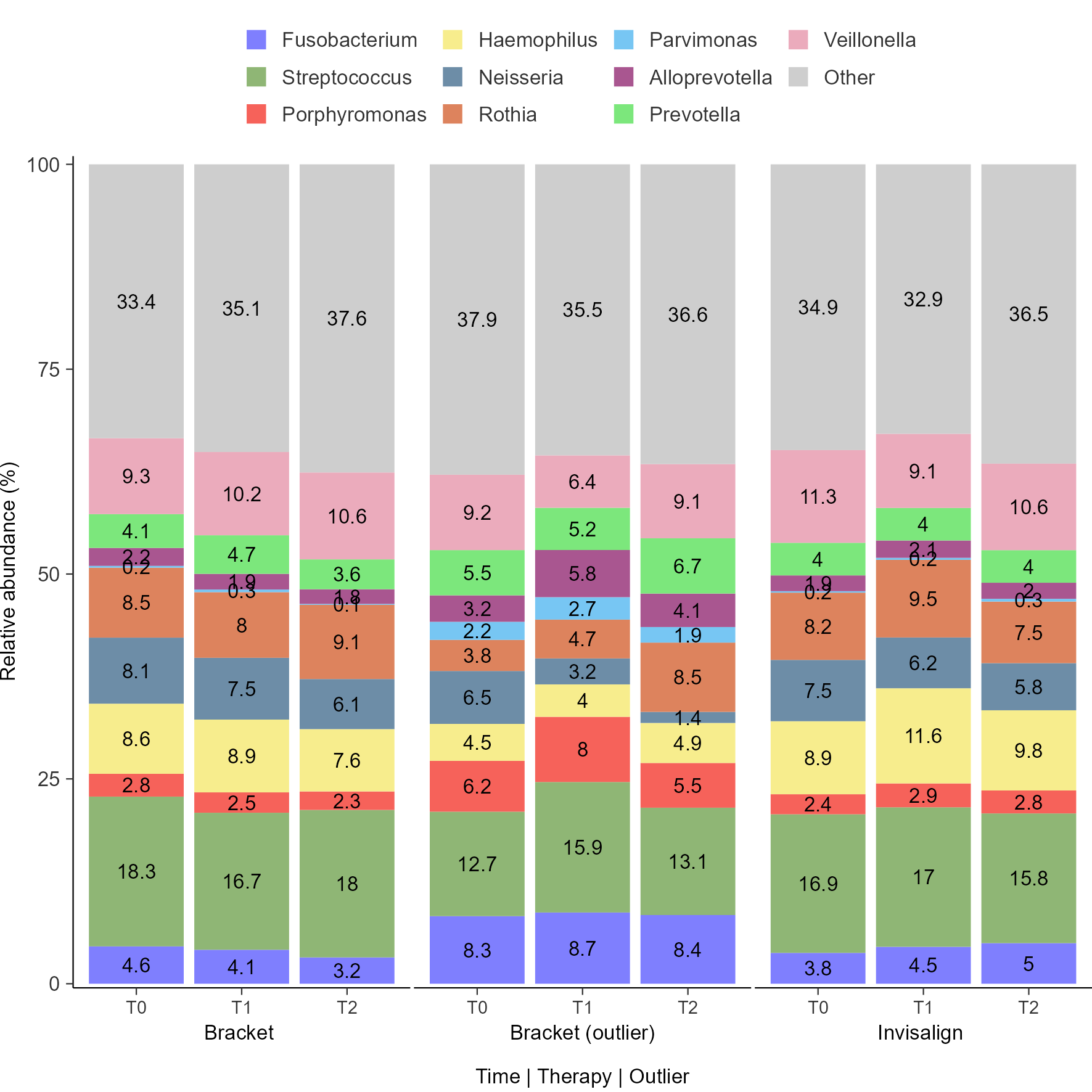

Supplement: Supplementary file 2 — Supplemental Fig.2. Relative abundance (%) of the top ten genera with the highest differences; Depicted are the groups Bracket, Bracket (Outliers) including information of patients excluded from MRP-8/14 analysis, and Invisalign. [file 40510_2025_560_MOESM2_ESM.png]

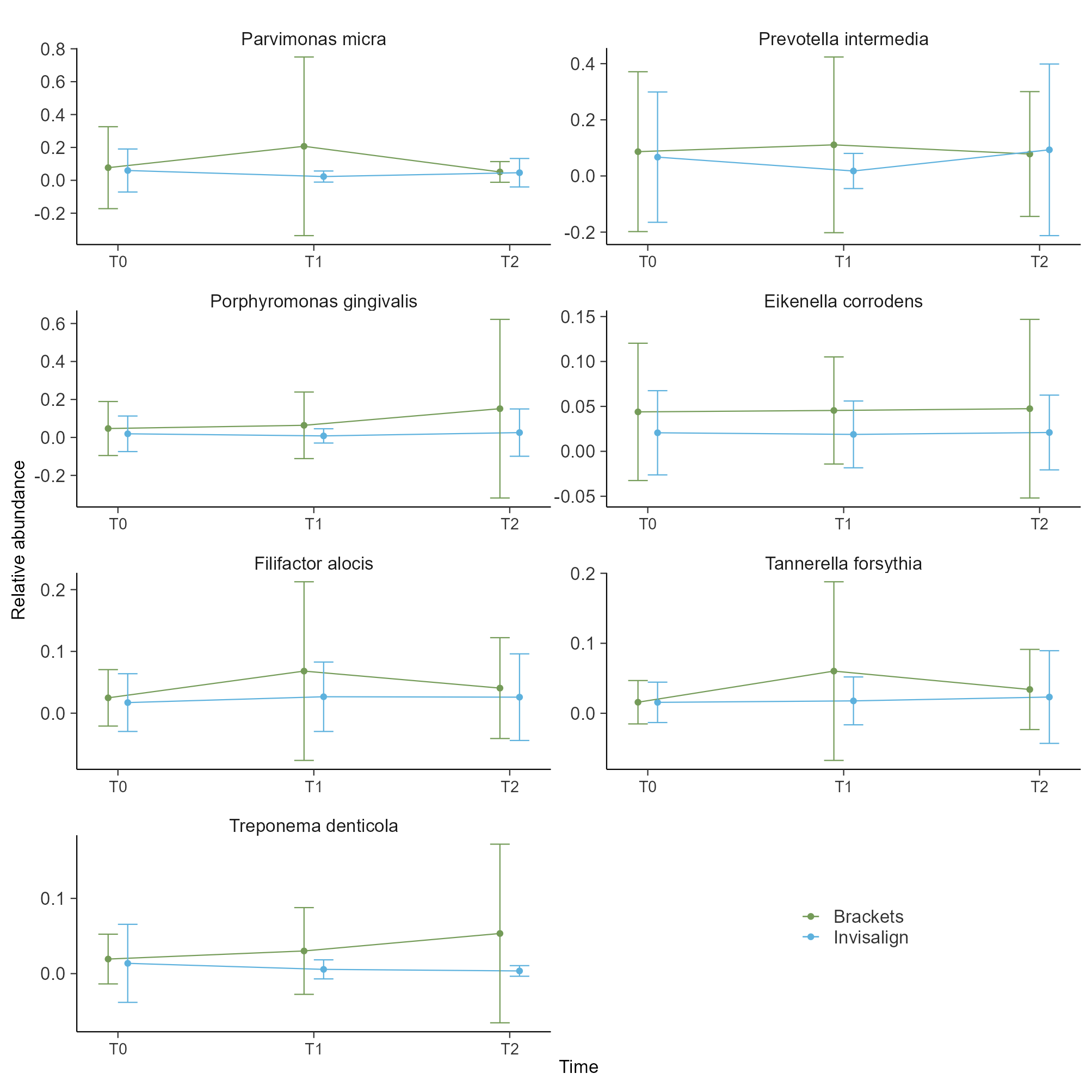

Supplement: Supplementary file 3 — Supplemental Fig.3 Variations in the relative abundance of gingivitis-relevant bacterial species; Depicted in the figure are relative abundance of Fusobacterium nucleatum, Parvimonas micra, Prevotella intermedia, Porphyromonas gingivalis, Eikenella corrodens, Filifactor alocis, Tannerella forsythia and Treponema denticola —over the three time points (T0, T1, and T2). The data from brackets (green lines) and Invisalign (blue lines) are depicted separately. Error bars indicate standard deviations from the mean. [file 40510_2025_560_MOESM3_ESM.png]
